# Electrostatic generator enhancements for powering IoT nodes via efficient energy management

**DOI:** 10.1038/s41378-024-00660-1

**Published:** 2024-03-06

**Authors:** Zibo Wu, Zeyuan Cao, Junchi Teng, Rong Ding, Jiani Xu, Xiongying Ye

**Affiliations:** State Key Laboratory of Precision Measurement Technology and Instruments, Department of Precision Instrument, Tsinghua University, 100084 Beijing, China

**Keywords:** Electrical and electronic engineering, NEMS

## Abstract

Electrostatic generators show great potential for powering widely distributed electronic devices in Internet of Things (IoT) applications. However, a critical issue limiting such generators is their high impedance mismatch when coupled to electronics, which results in very low energy utilization efficiency. Here, we present a high-performance energy management unit (EMU) based on a spark-switch tube and a buck converter with an RF inductor. By optimizing the elements and parameters of the EMU, a maximum direct current output power of 79.2 mW m^-2^ rps^-1^ was reached for a rotary electret generator with the EMU, achieving 1.2 times greater power output than without the EMU. Furthermore, the maximum power of the contact-separated triboelectric nanogenerator with an EMU is 1.5 times that without the EMU. This excellent performance is attributed to the various optimizations, including utilizing an ultralow-loss spark-switch tube with a proper breakdown voltage, adding a matched input capacitor to enhance available charge, and incorporating an RF inductor to facilitate the high-speed energy transfer process. Based on this extremely efficient EMU, a compact self-powered wireless temperature sensor node was demonstrated to acquire and transmit data every 3.5 s under a slight wind speed of 0.5 m/s. This work greatly promotes the utilization of electrostatic nanogenerators in practical applications, particularly in IoT nodes.

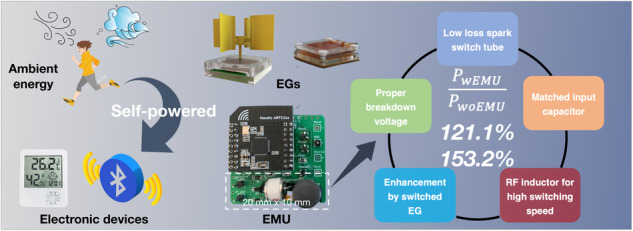

## Introduction

With the rapid development of the Internet of Things (IoT), there is an increasing demand for harvesting ambient energy to power billions of distributed sensor nodes^1^. Among the various energy harvesting technologies, electrostatic generators (EGs), including triboelectric nanogenerators^2,3^ and electret generators^4,5^, have been demonstrated to be promising for harvesting energy for low-power devices from wind, waves, environmental vibration, human motion, and more^1,6–8^ because of their high efficiency, lightweight, low cost, flexibility, and environmental adaptability, especially from low-frequency motion^9,10^.

However, a critical issue for EGs is their high output impedance and the corresponding impedance mismatch when coupled to conventional electronics and energy storage devices, resulting in very low efficiency when directly powering electronic devices. Recently, various types of energy management units (EMUs) have been proposed to reduce mismatches and improve the ability of EGs to power electronic devices^11–15^. Among them, EMUs with buck converters have been widely used and studied due to their high efficiency and simple structure^16,17^. For this type of EMU, switching is the key component for realizing high efficiency and reliability, and the on-resistance, off-resistance, parasitic capacitance, and conducting time greatly affect the performance of the EMU. Many types of switches have been proposed, such as electronic switches^17,18^, mechanical contact switches^19,20^, electrostatic vibration switches^21^, and discharge switches^22–24^. Among the various discharge switches, the spark switch is a type relatively ideal switch with low on-resistance, ultrahigh off-resistance, and no additional power supply or mechanical contacts^24^. In addition, optimizing the inductor in a buck converter can further enhance the performance of EMUs^25^. To evaluate the performance of EMUs, the ratio of the maximum power on a resistive load with an EMU to that without an EMU is utilized, which reflects the ability of an EMU to extract energy from an EG. The highest reported ratio is 97.1%^26^. However, because the output of an EG with a switch can be enhanced several times by realizing the cycle of maximum energy output (CMEO)^16,27^, the generator with an EMU featuring a switch can theoretically obtain an even greater output than its direct output. Unfortunately, due to the relatively high energy losses in previous EMUs, such a result has yet to be demonstrated. Therefore, there is still much room for enhancing the capability of EGs for powering electronic devices by improving the performance of EMUs.

Here, a high-performance EMU based on an LC passive buck converter is implemented through optimization, including utilizing an ultralow-loss spark-switch tube (SST) with a proper breakdown voltage, adding a matched input capacitor to enhance the available charge, and incorporating an RF inductor to facilitate high-speed energy transfer. By optimizing the elements and parameters of the EMU, the maximum average power with the EMU reaches 1.2 times that without the EMU for a rotary electret generator (REG), realizing a DC power density of 79.2 mW m^-2^ rps^-1^. In addition, as a contact-separation triboelectric nanogenerator (CS-TENG), the maximum average power with an EMU is 1.5 times that without an EMU. Benefiting from the excellent performance of the proposed EMU, a self-powered wireless temperature sensor is demonstrated. At a slight wind speed of 0.5 m/s, the sensor can complete environmental temperature data acquisition and wireless transmission every 3.5 s. The EMU proposed in this work significantly enhances the DC output capacity of the electrostatic generator, which will strongly promote practical applications of the electrostatic generator.

## Results and discussion

**Fig. 1:**
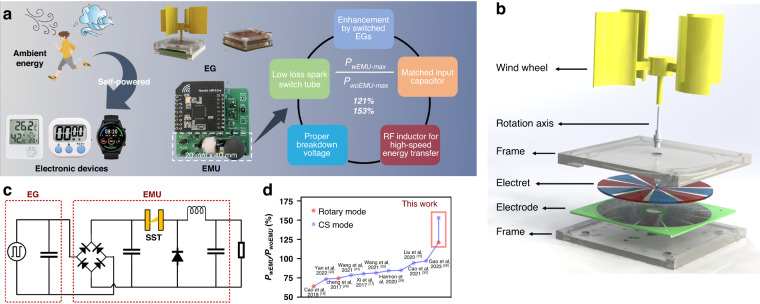
Design and performance of the EMU for EGs to power an IoT node. **a** Illustration of EGs with a remarkably good performance in harvesting ambient energy to power electronic devices. **b** Structural diagram of REG for wind energy harvesting. **c** Circuit schematic of the EMU for REG. **d** Ratio of the maximum power with an EMU to that without an EMU in this work compared with that reported previously

To further promote the applications of EGs in IoT nodes, a high-performance EMU for EGs to harvest ambient motion energy is developed here (Fig. 1). The EMU comprises a rectifier, an input capacitor, an SST, and a buck converter with an RF inductor. By determining the suitable ultralow-loss SST with a proper breakdown voltage, adding a matched input capacitor, and combining an RF inductor to facilitate high-speed switching, our EMU achieves remarkable performance. With the EMU, a REG gains a DC output power of 79.2 mW m^-2^ rps^-1^ at the optimal load, which is 1.2 times greater than the output power of the generator without the EMU. Furthermore, this value is 1.5 times that of the CS-TENG. This represents a novel demonstration of a generator with an EMU that achieves a higher output power than does the original generator without an EMU (Table S1)^15–17,23–26,28–30^.

In this paper, we first demonstrate the power enhancement of REG with a spark switch and input capacitor and analyze the enhancement effects of the breakdown voltage of the spark switch and the input capacitor on the output performance of a switched REG (swREG). Second, we compared the discharge characteristics of different types of commercial spark switches and found that the SST had the best performance. Third, we optimized the EMU, including the breakdown voltage of the SST, the capacitance of the input capacitor, and the type of inductor in the EMU, and obtained a high-performance EMU through experiments. Ultimately, with this high-performance EMU, a self-powered wireless temperature sensor node with an REG was demonstrated.

### Power enhancement of REG with spark switch and input capacitor

**Fig. 2:**
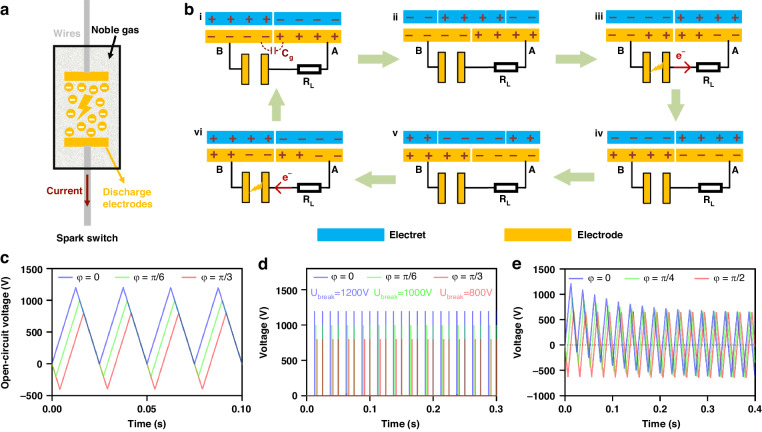
Working mechanisms of a spark switch and swREG. **a** Working mechanism of a spark switch. **b** Working process of swREG. **c** Simulated open-circuit voltages of REG 1000 TΩ under different *φ*. **d** Output voltages of swREG under a load of 1 KΩ for different *φ*. **e** Output voltages of REG on a load of 10 GΩ

As a simple and autonomous high-voltage switch, the spark switch is suitable for EMUs; its relevant working mechanism is shown in Fig. 2a. Typically, a spark switch consists of discharge electrodes and noble gas enclosed in a ceramic or glass tube. When the voltage across the switch exceeds its breakdown voltage (±*U*_*break*_), the noble gas breaks down, providing a path for electric current discharge. To clarify the effect of the spark switch on the power enhancement for EGs, we investigate a swREG that is formed by connecting a spark switch with a bipolar-charged REG. Supposing *U*_*break*_ equal to the maximum voltage of the swREG, the swREG’s working process can be described as shown in Fig. 2b: (i) in the initial state, the negatively charged electret and positively charged electret are aligned with Electrode A and B, respectively, *U*_*AB*_ is zero and the switch is off; (ii) as the rotor rotates, *U*_*AB*_ gradually increases; (iii) when *U*_*AB*_ increases to the breakdown voltage of the spark switch, the switch is turned on and the energy in REG releases to the small load; (iv) then, *U*_*AB*_ returns to zero and the switch is turned off; (v) as the rotor rotates, *U*_*AB*_ gradually increases from 0 to -*U*_*break*_; (vi) finally, when *U*_*AB*_ reaches -*U*_*break*_, the switch is turned on again and the energy releases to the resistor, such that *U*_*AB*_ returns to zero.

In this process, the spark switch’s breakdown moment is the most important condition, as the energy transfer from the generator to the load occurs at this time. The transferred energy can be approximately calculated as ${E}_{{cg}}=\frac{1}{2}{C}_{g}{U}_{{break}}^{2}$, in which *C*_*g*_ is the capacitor of the generator formed by the electrode pairs. Thus, to obtain more energy, *U*_*break*_ should be as close as possible to the maximum absolute value of the open-circuit voltage (*U*_*peak*_) of REG, which varies with the initial phase *φ* of REG (Fig. 2c). The output voltages of the swREG with *U*_*break*_ = *U*_*peak*_ after a rectifier on a load of 1 KΩ are shown in Fig. 2d. As shown, the output energy of swREG also varies with the initial phase *φ* of REG and reaches the highest value at *φ* = 0, which is the case shown in Fig. 2b. The initial phase *φ* is defined as the phase difference between the electret and electrode at the moment the REG starts operation (Fig. S1).

In general, *U*_*peak*_ decreases gradually to the same stable value, even with any *φ* (Fig. 2e), when the resistance between Electrodes A and B is not sufficiently large; i.e., there is a nonideal open circuit. This stable value is the commonly measured open-circuit voltage of REG^31^, which is equal to the minimum *U*_*peak*_ of REG that is at $\varphi =\pi /2$ (Fig. S2). Here, Fig. 2c–e are obtained by simulation using LT-SPICE (details in Note S1). We define the minimum *U*_*peak*_ as *U*_*oc*_, which can be expressed by the following equation from our previous work^32^:

1 $${U}_{{oc}}=\frac{\sigma S}{2{C}_{g}}$$

where *S* is the area of the electret and *σ* is the charge density of the electret. As shown in Fig. 2d, *φ* has a great effect on the extractable energy from swREG, and we can choose *U*_*break*_ ≤ 2*U*_*oc*_ to make the spark switch work at some *φ*. However, to guarantee that the spark switch can work at any *φ*, *U*_*break*_ must satisfy *U*_*break*_ ≤ *U*_*oc*_. When *U*_*break*_ = *U*_*oc*_, the charge released to the load is *U*_*oc*_*C*_*g*_, equal to *σS/2* from Eq. (1); i.e., only half of the charge is utilized, signifying that the energy of swREG cannot be fully transferred to the external load. To fully utilize this charge, we add an input capacitor *C*_*in*_ to the rectifier bridge in parallel to temporarily store a portion of the energy from the REG (Fig. S3). In this connection, *C*_*in*_ should satisfy the following equation:

2 $$\frac{\sigma S}{{C}_{g}+{C}_{{in}}}\ge {U}_{{oc}}\Rightarrow {C}_{{in}}\le {C}_{g}$$

Then, the energy released from swREG for one discharge is:

3 $${E}_{{in}}=\frac{1}{2}({C}_{g}+{C}_{{in}}){U}_{{break}}^{2}$$

To maximize *E*_*in*_, *C*_*in*_ should be equal to *C*_*g*_. When *C*_*in*_ = *C*_*g*_ and *U*_*break*_ = *U*_*oc*_, the average power of swREG can reach its maximum as follows:

4 $${P}_{{sw}-\max }{=1.89\times P}_{{woEMU}}$$

where *P*_*woEMU*_ is the maximum average power on the matched load without an EMU. Namely, the maximum average power of REG with a threshold-voltage switch that can work at any initial phase is 1.89 times as high as the matched average power of the common REG in theory (details in Note S2), which is 1/2 of that with a synchronous switch^16^. This result indicates that the output power of the swREG can be largely enhanced by properly setting *U*_*break*_ and *C*_*in*_.

### Optimization of spark switch

**Fig. 3:**
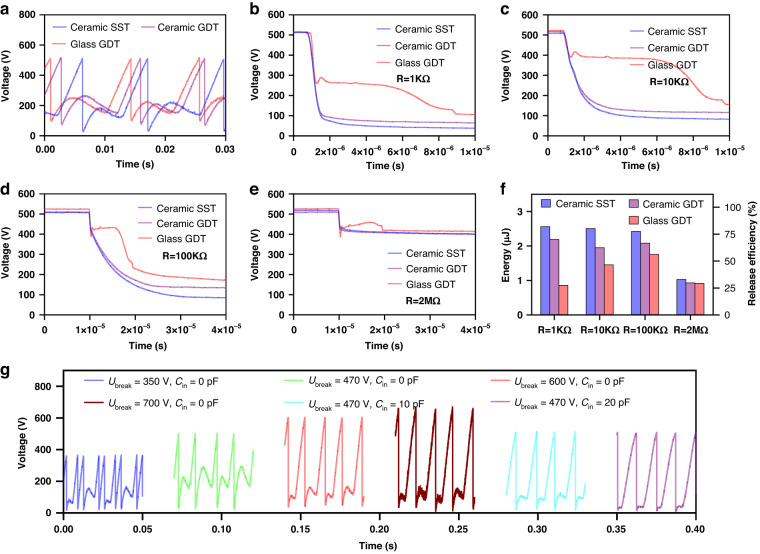
Discharge characteristics of three types of spark switches. **a** Measured *U*_*ab*_ without *C*_*in*_. **b**–**e** *U*_*ab*_ around the breakdown point under a load of 1 KΩ ~ 2 MΩ. **f** Obtained energy in one discharge and release efficiency of spark switches under different loads. **g** *U*_*ab*_ respect to the SST under different *U*_*break*_ and *C*_*in*_

As the key component in swREG, the characteristics of the switch strongly affect the performance of swREG. To identify the spark switch with the best performance, we selected three types of commercial spark switches—a glass gas-discharge tube (glass GDT), a ceramic gas-discharge tube (ceramic GDT), and a ceramic SST (Fig. S4)—and experimentally compared their discharge characteristics based on a REG. A bipolar-charged electret generator with a 5 cm diameter (Fig. S5) was used in the experiment, and the structural parameters of the generator are listed in Table S2. The open-circuit voltage and short-circuit current of the REG are shown in Fig. S6, where *U*_*oc*_ is 520 V and does not change with respect to the rotation speed. The glass GDT, ceramic GDT, and ceramic SST with nominal *U*_*break*_ values of 500 V, 470 V, and 470 V are used due to the limitations of the choice. We measured the voltage across the rectifier before the spark switch (*U*_*ab*_) at the discharge points of these spark switches under different load resistances (Fig. S7). Figure 3a shows the *U*_*ab*_ under a load of 1 KΩ. It is clear that all the spark switches breakdown at a voltage of approximately 510 V, but *U*_*ab*_ for the SST after breakdown is the lowest and close to zero, indicating that the SST releases the most energy. The *U*_*ab*_ values around the breakdown point under a load of 1 KΩ ~ 2 MΩ are shown in Fig. 3b–e. It is clear that the discharge rates of ceramic GDT and SST are significantly faster than those of glass GDT. The energy in the *C*_*g*_ is released more thoroughly by SST, indicating that the SST has the lowest loss of switching. The voltages on a load of 1 KΩ –2 MΩ at the breakdown point are shown in Fig. S8. By integrating the discharge curves in Fig. S8, the energies obtained for the load *E*_*load*_ in one discharge cycle and the energy release efficiencies of the spark switches are shown in Fig. 3f. Here, the release efficiency is defined as:

5 $${\eta }_{{sw}-{re}}=\frac{{E}_{{load}}}{{E}_{{cg}}}\times 100 \% =\frac{{E}_{{load}}}{\frac{1}{2}{C}_{g}{U}_{{break}}^{2}}\times 100 \%$$

where *U*_*break*_ = 510 V, and *C*_*g*_ = 25 pF measured by a precision LCR meter. It is concluded that the SST exhibits its highest efficiency (82.2%) under a load of 1 KΩ, and the efficiency remains almost unchanged until the load is increased to 100 KΩ. The efficiencies at a 2 MΩ load for the three spark switches are much lower than those at lower resistance, which may result from incomplete discharge due to the ultrashort breakdown period (Fig. 3e). The voltages on a load for a long time are shown in Fig. S9, wherein the SST exhibits the best long-term stability and remains a constant output after 240,000 cycles. In summary, the SST has the best performance and was chosen for subsequent experiments.

To evaluate the influence of *U*_*break*_ and *C*_*in*_ on the swREG working process, we measured *U*_*ab*_ with SST under different *U*_*break*_ without *C*_*in*_ and under *U*_*break*_ = 470 V with different *C*_*in*_ (Fig. 3g). Clearly, the SST can be turned on when *U*_*break*_ = 700 V, which is much greater than the measured *U*_*oc*_. This effect is attributed to the following two reasons: (1) the switch can be turned on at some *φ* when *U*_*oc*_ ≤ *U*_*break*_ ≤ 2*U*_*oc*_ in theory; (2) due to the input resistance and parasitic capacitance of the measurement circuit for *U*_*oc*_, the measured *U*_*oc*_ is less than its true value, and the actual *U*_*oc*_ is 687 V (Note S3). In addition, the SST cannot be turned on under *U*_*break*_ = 800 V (Fig. S10A), which is lower than the theoretical maximum *U*_*break*_ (2*U*_*oc*_), mainly due to the leakage current of the rectifier and other nonideal conditions. Notably, the introduction of the *U*_*ab*_ measurement circuit decreases the usable values of *U*_*break*_ and *C*_*in*_, which should be larger in practical EMUs. Thus, *U*_*break*_ and *C*_*in*_ must be further optimized in EMUs to maximize the extractable energy from swREG.

### Optimization of EMUs

**Fig. 4:**
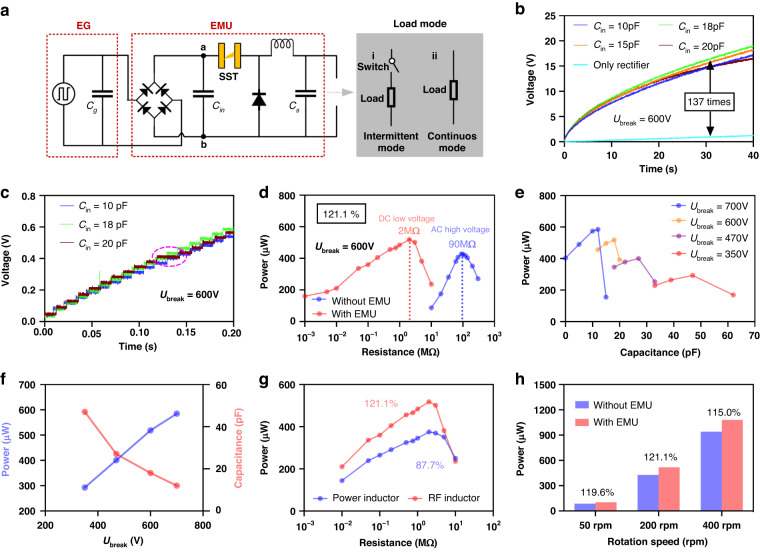
Performance of the EMU for the REG under different *U*_break_ and *C*_in_. **a** Schematic of the EMU in two working modes. **b**, **c** Charging curves for *C*_*s*_ = 100 μF under *U*_*break*_ = 600 V and a zoom-in in the first 0.2 s. **d** Power‒load curve under *U*_*break*_ = 600 V and *C*_*in*_ = 18 pF. **e** Maximum average power vs. *C*_*in*_ under different *U*_*break*_. **f** Maximum average power and the matched *C*_*in*_ vs. *U*_*break*_. **g** Comparison of the power-load curves for the power inductor and RF inductor and **h** Maximum average power at different rotation speeds for Ubreak = 600 V and Cin = 18 pF

To convert the high-voltage pulse output to a low-voltage DC output, we used a buck converter that consisted of a diode, an inductor, and a storage capacitor *C*_*s*_ combined with a rectifier and SST, to form an EMU (Fig. 4a). With an EMU, there are two types of working modes for practical applications: the intermittent mode and continuous mode. For the intermittent mode, the energy is temporarily stored in a relatively large capacitor *C*_*s*,_ and the load is driven only when the energy on *C*_*s*_ is sufficient (Fig. 4a-i). For the continuous mode, the energy powers the load continuously, and the capacitance of *C*_*s*_ is typically low, serving to stabilize the voltage on the load (Fig. 4a-ii). Figure S11A, B show the charging curves on *C*_*s*_ in the intermittent mode and the power-load curves in the continuous mode for the three types of spark switches, respectively, in which the SST shows the highest performance.

With an EMU, the energy stored in *C*_*in*_ and *C*_*g*_ first transfers to the inductor through the switch and subsequently transfers to *C*_*s*_ from the inductor. The characteristics of the inductor, which is one of the core components of an EMU, also greatly affect its efficiency. Considering the high switching speed feature of SSTs, power inductors commonly used in buck converters might not be the best fit. Here, we compared two types of inductors: the RF inductor, which has a higher self-resonant frequency and is usually used for high-frequency applications; and the power inductor, which has a higher-rated DC current, can store more energy and is typically used in EMUs. The experimental results are shown in Note. S4. The performance of the EMU with the RF inductor is better than that with the power inductor. This is attributed to the power inductor not responding rapidly enough to support the high-speed energy transfer process, resulting in a greater energy loss than occurs with the RF inductor. Based on this improved performance, RF inductors were used.

To optimize *U*_*break*_ and *C*_*in*_ for maximizing the performance of EMUs in both intermittent and continuous modes, the outputs of EMUs with different *U*_*break*_ and *C*_*in*_ values were measured. Figure 4b shows the charging curves for *C*_*s*_ = 100 μF under *U*_*break*_ = 600 V with different *C*_*in*_ values, and Fig. 4c shows a magnified view of the initial periods. With the optimal *C*_*in*_ = 18 pF, the charging power with an EMU is 137 times greater than that achieved by direct charging with a rectifier. The energy transfer efficiency of the EMU in charging the storage capacitor reaches approximately 85% at the stable stage (details in Note. S5). Theoretically, when *C*_*in*_ is larger, the extractable energy is greater when *C*_*in*_ is smaller than *C*_*g*_ = 25 pF, but actually, the energy is lower when *C*_*in*_ = 20 pF. This occurs because SSTs with *C*_*in*_ = 20 pF can sometimes not be turned on, as shown in the pink circle in Fig. 4c, which is attributed to the drifting of SSTs’ *U*_*break*_, resulting in the voltage accumulating in the larger *C*_*in*_ in a cycle lowering *U*_*break*_. Moreover, the effect of the initial phase *φ* of REG on the charging performance of EMUs with different *U*_*break*_ and *C*_*in*_ values was also studied (Fig. S12). With *U*_*break*_ = 600 V and *C*_*in*_ ≤ 18 pF, the SST can be turned on at any *φ*, and when *U*_*break*_ = 700 V and *C*_*in*_ = 0, the SST can be turned on at most *φ* but cannot be turned on at a few *φ*. The charging curves with *U*_*break*_ = 700 V under different *C*_*in*_ values are shown in Fig. S13A when the SST can be turned on.

The average power at different loads with the optimal *C*_*in*_ under *U*_*break*_ = 600 V and 700 V are shown in Fig. 4d and Fig. S13B, respectively, compared with the average power without an EMU. The average power at a load *R* is obtained from *U*_*rms*_^*2*^*/R* by measuring the root-mean-square of the voltage on the load. With the EMU, maximum output power densities of 79.2 mW m^-2^ rps^-1^ and 98.4 mW m^-2^ rps^-1^ are obtained, corresponding to 121.1% and 136.7% of that without EMU (65.4 mW m^-2^ rps^-1^), for *U*_*break*_ = 600 V and 700 V, respectively, from the following equation:

6 $${P}_{{ratio}}=\frac{{P}_{w{EMU}}}{{P}_{{woEMU}}}\times 100 \%$$

where *P*_*wEMU*_ is the maximum average power on the load matched with the EMU. Moreover, the efficiency *η*_*e*_ of the energy transferred from *C*_*g*_ and *C*_*in*_ to the matched load through the buck converter with an SST can be defined as follows:

7 $${\eta }_{e}=\frac{{P}_{{wEMU}}T}{{E}_{{in}}}\times 100 \% =\frac{{P}_{{wEMU}}T}{\frac{1}{2}({C}_{g}+{C}_{{in}}){U}_{{break}}^{2}}\times 100 \%$$

where *T* is the working period of the rectified swREG and is 12.5 ms here. *η*_*e*_ are 83.9% and 80.8% for *U*_*break*_ = 600 V and 700 V, respectively. In addition, *P*_*wEMU*_ increases with *U*_*break*_ under their optimal *C*_*in*_, and the optimal *C*_*in*_ decreases with *U*_*break*_ (Fig. 4e, f). Because, when *U*_*break*_ = 700 V, the SST cannot be turned on at some *φ*, the optimal parameters of the EMU are *U*_*break*_ = 600 V and *C*_*in*_ = 18 pF. Furthermore, we compared the RF inductor and power inductor both with 4.7 mH for the EMU. The performance of the EMU with the RF inductor is much better, as shown in Fig. 4g and Fig. S14, which is consistent with the experimental results in Note. S4. In addition, the maximum average power under different rotation speeds is shown in Fig. 4h (details in Fig. S15), indicating that the EMU has high performance over a wide operating frequency range.

**Fig. 5:**
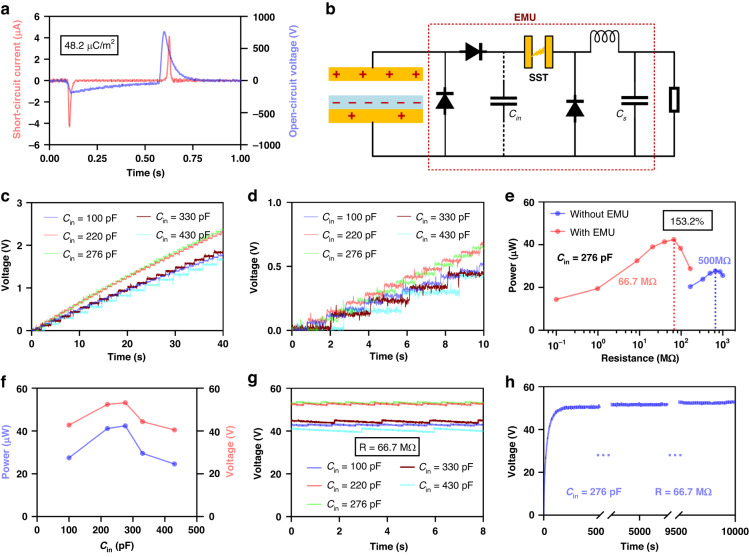
Performance of the EMU for a CS-TENG under different *C*_*in*_ with *U*_*break*_ = 600 V. **a** Short-circuit current and open-circuit voltage of the CS-TENG. **b** Schematic of the EMU for a CS-TENG. **c**, **d** Charging curves for *C*_*s*_ = 100 μF and their enlarged views. **e** Power-load curve under the optimal *C*_*in*_ = 276 pF. **f** Maximum average power and voltage on the matched load. **g** Curves of the voltage on the matched load. **h** Long-term stability of the CS-TENG with the EMU

To verify the universal powering capabilities of the EMU, the performance of the EMU for a CS-TENG was also studied. Here, a 4 cm × 4 cm CS-TENG (Fig. S16) was used, and the corresponding open-circuit voltage and short-circuit current are shown in Fig. 5a. The CS-TENG was driven by a linear motor at a working period of 1 Hz, and the maximum separation distance was 1 mm. A half-wave rectifier and an SST with *U*_*break*_ = 600 V are used in the EMU for the CS-TENG (Fig. 5b). In the intermittent mode, the charging curves for *C*_*s*_ = 100 μF under different *C*_*in*_ are shown in Fig. 5c, and their zoom-in is shown in Fig. 5d. The SST is turned on once per cycle when *C*_*in*_ ≤ 276 pF, but it is turned on once per every two or three cycles when *C*_*in*_ = 330 pF or 430 pF. In continuous mode, the power-load curve under *C*_*s*_ = 1 μF with the optimal *C*_*in*_ = 276 pF is shown in Fig. 5e, compared with that without an EMU. The maximum average power with the EMU is 153.2% of that without an EMU, with an output energy density of 26.5 mJ m^-2^ cycle^-1^. In addition, the power-load curves with different *C*_*in*_ are shown in Fig. S17. The maximum average powers and output voltages on the matched load with different *C*_*in*_ are shown in Fig. 5f. Figure 5g shows the curves of the output voltages on the matched load, exhibiting the same turn-on statuses with Fig. 5d. The CS-TENG with the EMU demonstrates good long-term stability and the output doesn’t drop after working for 10,000 cycles at 1 Hz (Fig. 5h). Here, the inductor of the EMU for the CS-TENG is an RF inductor for high current, which is appropriate for transferring more energy in one cycle than that for REG.

### Application demonstration

**Fig. 6:**
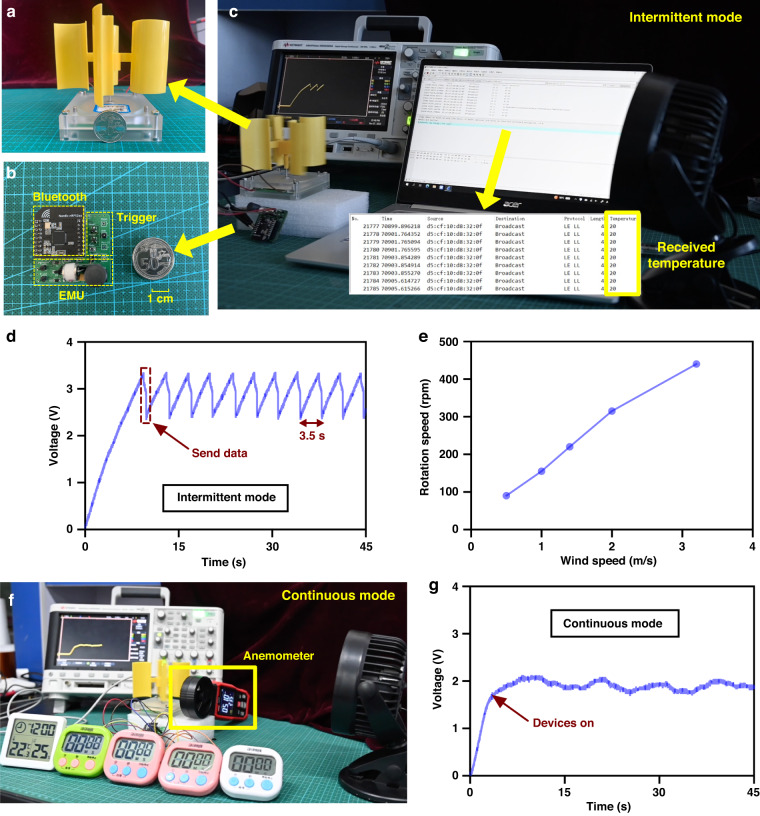
Application demonstrations of an REG with the EMU. **a** Photo of the REG with a wind wheel. **b** Circuit of the wireless sensor node. **c** Working scene of the wireless sensor node. **d** Voltage of the Bluetooth transmitter at a wind speed of 0.5 m/s in intermittent mode. **e** Relationship between the wind speed and rotation speed of the REG. **f** Working scene for powering a thermohygrometer and four timers in parallel under a wind speed of 0.5 m/s in continuous mode, and **g** the voltage on the devices

Based on the remarkable performance of the EMU, we demonstrated a self-powered wireless temperature sensor node driven by a 5 cm diameter REG with a small wind wheel with a 10 cm diameter (Fig. 6a). The circuit of the sensor node is shown in Fig. 6b; this circuit consists of an EMU, a trigger circuit (Fig. S18), and a Bluetooth transmitter with a temperature sensor, which works in intermittent mode. When the voltage on *C*_*s*_ reaches a certain level, the trigger circuit connects *C*_*s*_ to the wireless chip and powers it. At a slight wind speed of approximately 0.5 m/s, the self-powered wireless sensor node can send temperature data to the receiver every 3.5 s (Fig. 6c, d and Video S1). Moreover, the rotation speed of the REG scales linearly with the wind speed (Fig. 6e), demonstrating the possibility of simultaneously sensing temperature and wind speed, where the latter can be obtained from the transmission time interval. In this experiment, the wind speed was measured by an anemometer. In addition, a thermohygrometer with four timers in parallel can be continuously driven by the REG with an EMU in continuous mode at a wind speed of approximately 0.5 m/s (Fig. 6f and Video S2), and the voltage on the devices is shown in Fig. 6g. With this remarkably efficient EMU, EGs exhibit a great ability to drive a variety of practical electronics.

## Conclusion

In this work, a high-performance EMU based on a spark-switch tube and a buck converter with an RF inductor was presented. By utilizing an ultralow-loss spark switch with a proper breakdown voltage, adding a matched input capacitor, and combining an RF inductor to facilitate high-speed switching, a rotary electret generator with the optimized EMU experimentally achieved higher output power than the original generator, which is the first time. The maximum output power with the EMU was 121.1% and 153.2% of that without an EMU for a rotary electret generator and a contact-separation triboelectric nanogenerator, respectively, which achieved DC power/energy densities of 79.2 mW m^-2^ rps^-1^ and 26.5 mJ m^-2^ cycle^-1^. In addition, the EMU with a spark switch also exhibited great long-term reliability. Due to the excellent performance of the EMU, a self-powered wireless temperature sensor node was implemented, which acquired and transmitted the temperature data every 3.5 s under an ultralow wind speed of 0.5 m/s with a generator with a 5 cm diameter. The proposed EMU significantly enhances the DC output capacity of the electrostatic generator, which promotes practical applications of the electrostatic generator in IoT nodes.

## Experimental

### Fabrication of the REG

The PTFE film (HF-50, HongFu Co., Ltd.) was charged by the patterned contact microdischarge method at -4500 V for 5 min and +4500 V for 3 min, as described in our previous work^33^, and was stuck to a disk of 5 cm in diameter to form the rotor. The stator is fabricated by a PCB process on an FR4 substrate and assembled with the rotor. All three types of spark switches were purchased from Shenzhen Shaoxin Electronics Co., Ltd. The glass GDT, ceramic GDT, and ceramic SST types used were SSD35, SXH8, and KG8, respectively. The inductor used in the EMU is an RF inductor with 4.7 mH, ID 07MFG-472J-50 from Fastron Co., Ltd. The type of diode in the EMU is RFU02VS8SG. The power inductor used in the experiments was RFS1317-4.7 mH from Coilcraft Co., Ltd.

### Fabrication of the CS-TENG

An FPCB electrode film was stuck to an acrylic substrate, and then, a 30 µm thick PTFE film was stuck to the electrode using 15 µm thick double-sided adhesive polyethylene terephthalate tape to form the bottom part. Another FPCB electrode film is stuck to another acrylic substrate to form the top part. Finally, the top and bottom parts are assembled onto a linear motor for testing, as shown in Fig. S16. The maximum separation distance for the CS-TENG is 1 mm. The CS-TENG is precharged by the contact microdischarge method at –2000 V for 2 min when it is in contact, and it is precharged again when Cin is changed. The inductor in the EMU for the CS-TENG is an RF inductor with dimensions of 2.7 mH (ID 11PHC-272K-50 from Fastron Co., Ltd.).

### Measurement

The open-circuit voltage and *U*_*ab*_ were measured and captured by an oscilloscope (KEYSIGHT DSOX2024A, US) with a custom-made circuit, as described in our previous work^33^. The short-circuit current was measured by a digital multimeter (Keithley DMM7510, US) directly connected to the generator without an extra load. The rotation speed is calculated according to the frequency of the output voltage captured by an oscilloscope. *C*_*g*_ was measured by a precision LCR meter (TH 2816A, CN).

### Supplementary information

Revised supporting information

Video 1

Video 2
