# Supplementary material for: Electrostatic generator enhancements for powering IoT nodes via efficient energy management
Source: Microsyst Nanoeng. 2024 Mar 6;10:30. doi: 10.1038/s41378-024-00660-1 (PMC10918071; doi:10.1038/s41378-024-00660-1)
Supplement: Supplementary file 1 — Revised supporting information [file 41378_2024_660_MOESM1_ESM.pdf]

Supporting Information

Electrostatic generator enhancements for powering IoT nodes via efficient energy management

Zibo Wu, Zeyuan Cao*, Junchi Teng, Rong Ding, Jiani Xu and Xiongying Ye*

State Key Laboratory of Precision Measurement Technology and Instruments, Department of Precision Instrument, Tsinghua University, Beijing 100084, China

Table S1. Performance comparison of different EMU

**Reference**	**Type of EMU**	**Type of switch**	**Working mode**	$\frac{P_{wEMU}}{P_{woEMU}}$	**Published time**
[S1]	Buck converter	mechanical switch	Rotary	64.0%	2019
[S2]	Buck converter	film discharge switch	Contact-separation	73.6%	2022
[S3]	Flyback converter	MOSFET	Rotary	74.5%	2017
[S4]	Flyback converter	gas discharge tube	Contact-separation	78.5%	2021
[S5]	Buck converter	MOSFET	Contact-separation	80.4%	2017
[S6]	Buck converter	gas discharge tube	Contact-separation	81.6%	2021
[S7]	Buck converter	silicon-controlled rectifier	Contact-separation	84.3%	2020
[S8]	Buck converter	mechanical switch	Contact-separation	84.8%	2021
[S9]	Switched-capacitor-convertors	mechanical switch	Contact-separation	94.5%	2020
[S10]	Buck converter	mechanical switch	Contact-separation	97.1%	2023
This work	Buck converter	gas discharge tube	Rotary	**121.1%**	**/**
This work	Buck converter	gas discharge tube	Contact-separation	**153.2%**	**/**

Table S2. Structural parameters of the fabricated REG

Category	Parameters of generator	Value
**Rotor of REG**	Thickness of PTFE electret	30 μm
	Number of pairs of charged electret	12
	Outer radius of charged electret	25 mm
	Inner radius of charged electret	6 mm
**Stator of REG**	Number of pairs of electrodes	12
	Outer radius of electrodes	25 mm
	Inner radius of electrodes	6 mm
	duty ratio of electrodes	0.8
	Generator capacitance	25 pF
**Air gap**	Air gap between rotor and stator	0.15 mm

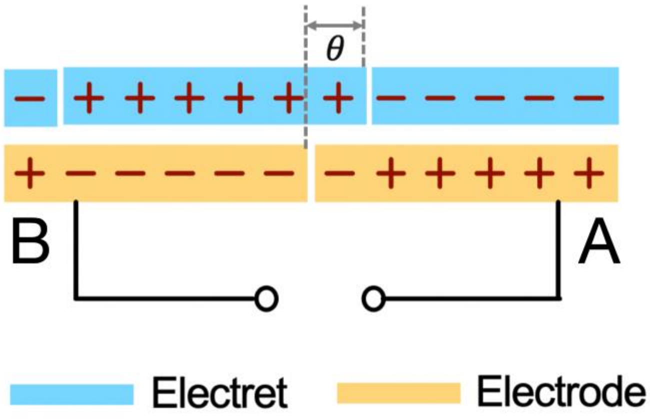

Figure S1. Definition of the initial phase. $\varphi = n\theta$, where n is the pairs of electrodes, θ is the angle difference between the positively charged electret and Electrode B at the moment of REG starting work.

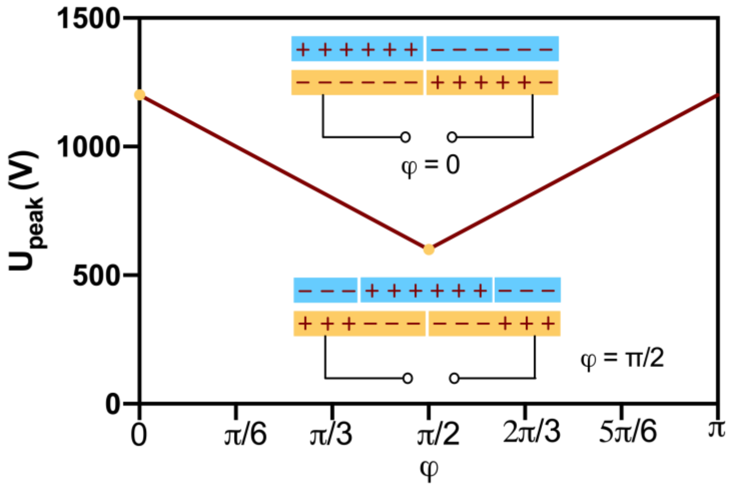

Figure S2. U_{peak} **under different** φ**.** Simulation details are shown in Note. S1.

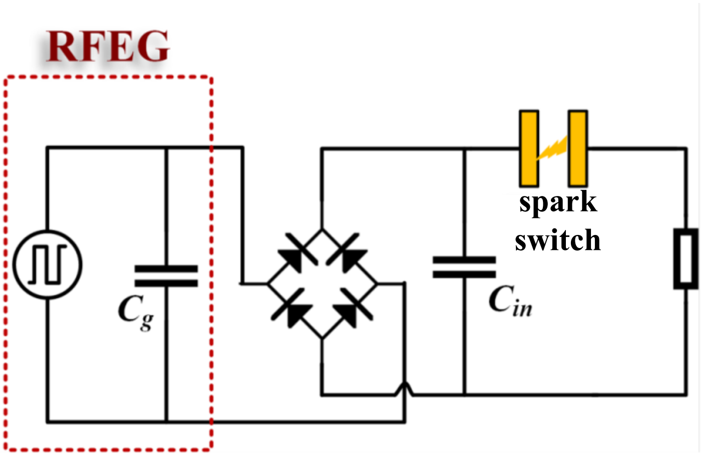

Figure S3. swREG with an input capacitor and a rectifier.

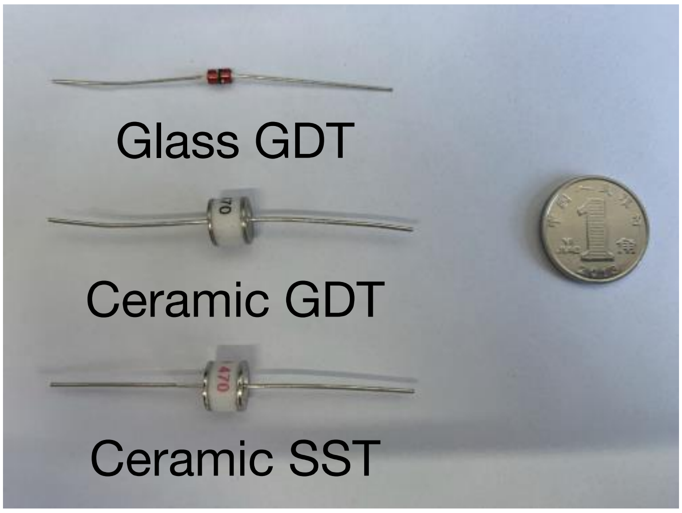

Figure S4. The photo of glass GDT, ceramic GDT and Ceramic SST. In general, Glass GDT and ceramic GDT are usually used for overvoltage protection, and ceramic SST is commonly used in the applications which need to generate high voltage pulses, and it has longer service lives.

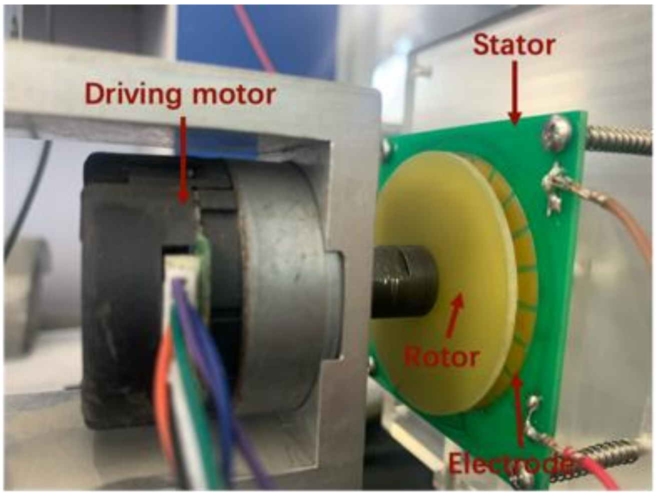

Figure S5. The photo of the fabricated REG.

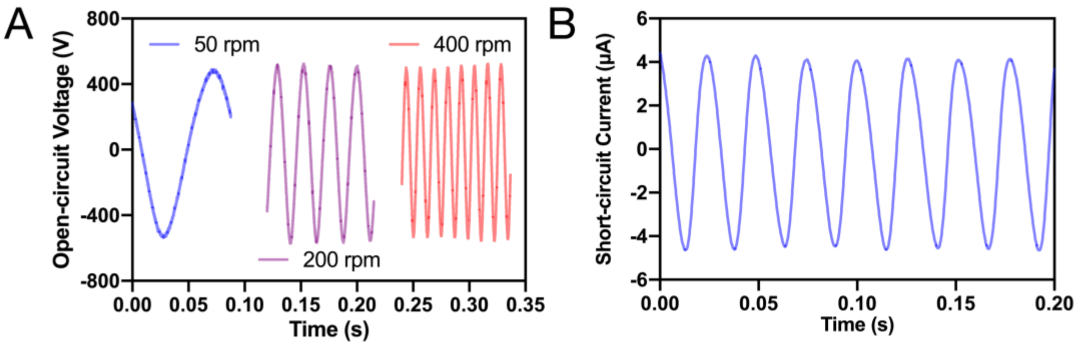

Figure S6. Output characteristics of the fabricated REG. (A) Open-circuit voltage of the fabricated REG under different rotation speeds. (B) Short-circuit current of the fabricated REG at 200 rpm.

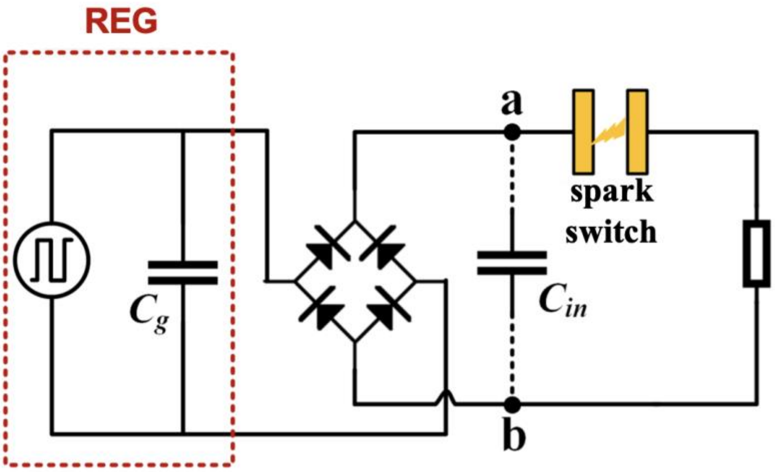

Figure S7. Schematic for the measured voltage cross the rectifier before the spark switch (U_{ab}). There is no C_{in} in the measurement circuit for Figure 3A~E.

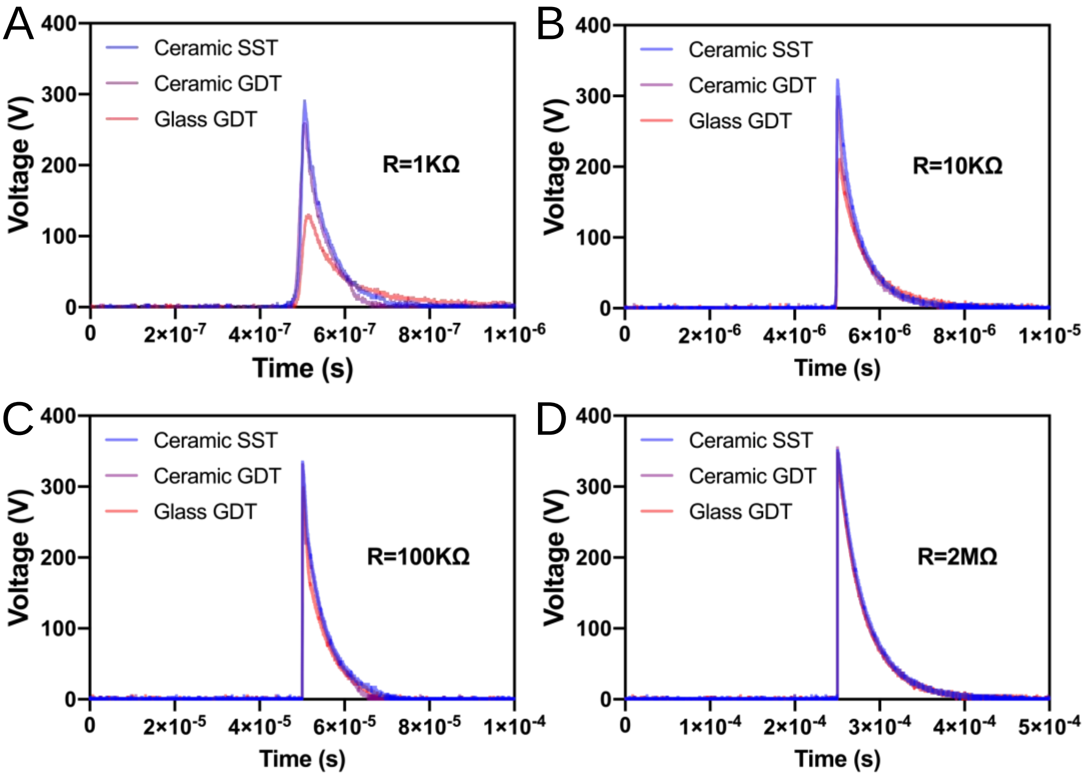

Figure S8. Output voltages on loads of 1 KΩ ~ 2 MΩ at the breakdown points with three spark switches. Apparently, the peak voltage on the loads with SST is the highest, indicating that SST has the lowest on-resistance. The peak value of the voltage on the load is much smaller than 510 V, since part of the released charges are transferred to the parasitic capacitance of the oscilloscope probe firstly, then to the load.

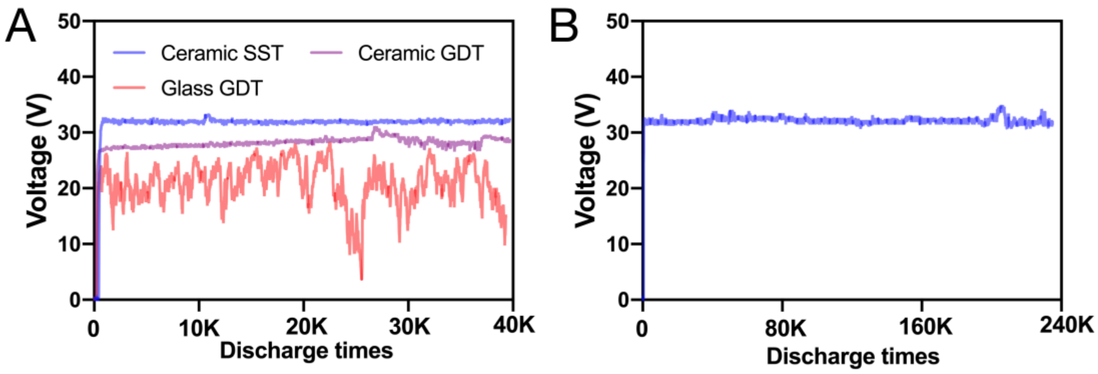

Figure S9. Results of longtime stability for three kinds of spark switches. (A) Longtime stability of the ceramic SST, ceramic GDT, and glass GDT in continuous mode with Cs = 1 μF and load of 2 MΩ. (B) Longtime stability of the ceramic SST in 240,000 times of discharges.

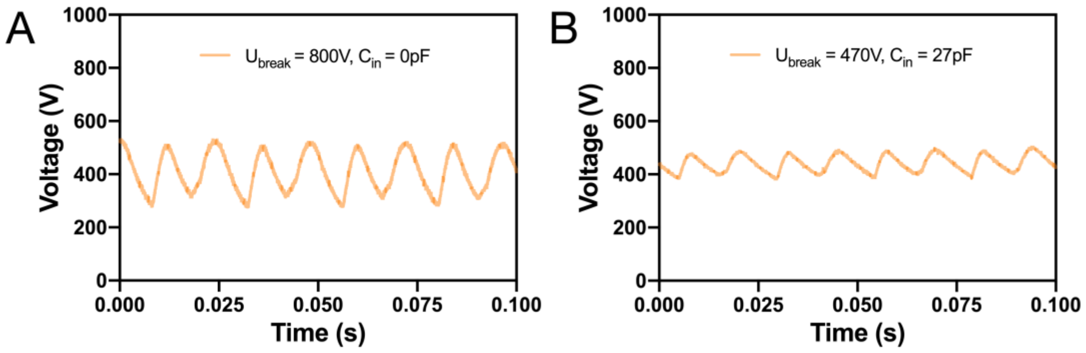

Figure S10. Measured U_{ab} under different conditions. (A) U_{ab} at U_{break} = 800 V without C_{in}. (B) U_{ab} at U_{break} = 470V with C_{in} = 27 pF.

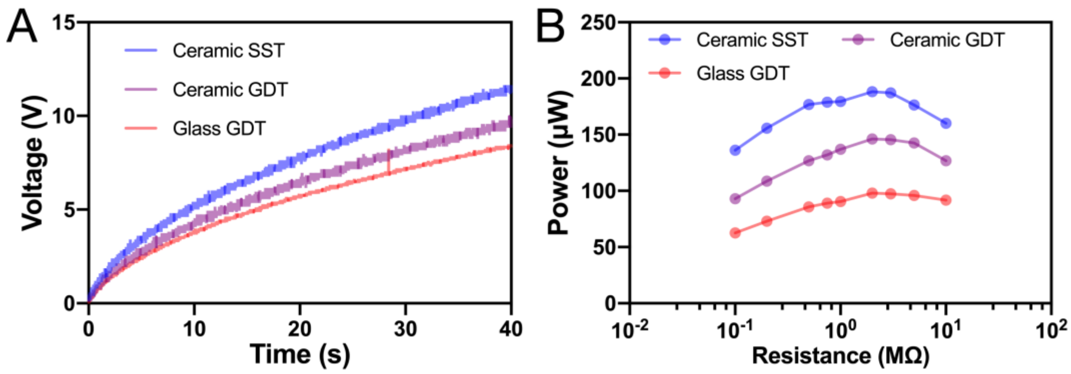

Figure S11. Measurement results of swREG under U_{break} = 470 V without C_{in}. (A) Charging curves on C_s = 100 μF in intermittent mode. (B) Power-load curves with C_s = 1 μF in continuous mode.

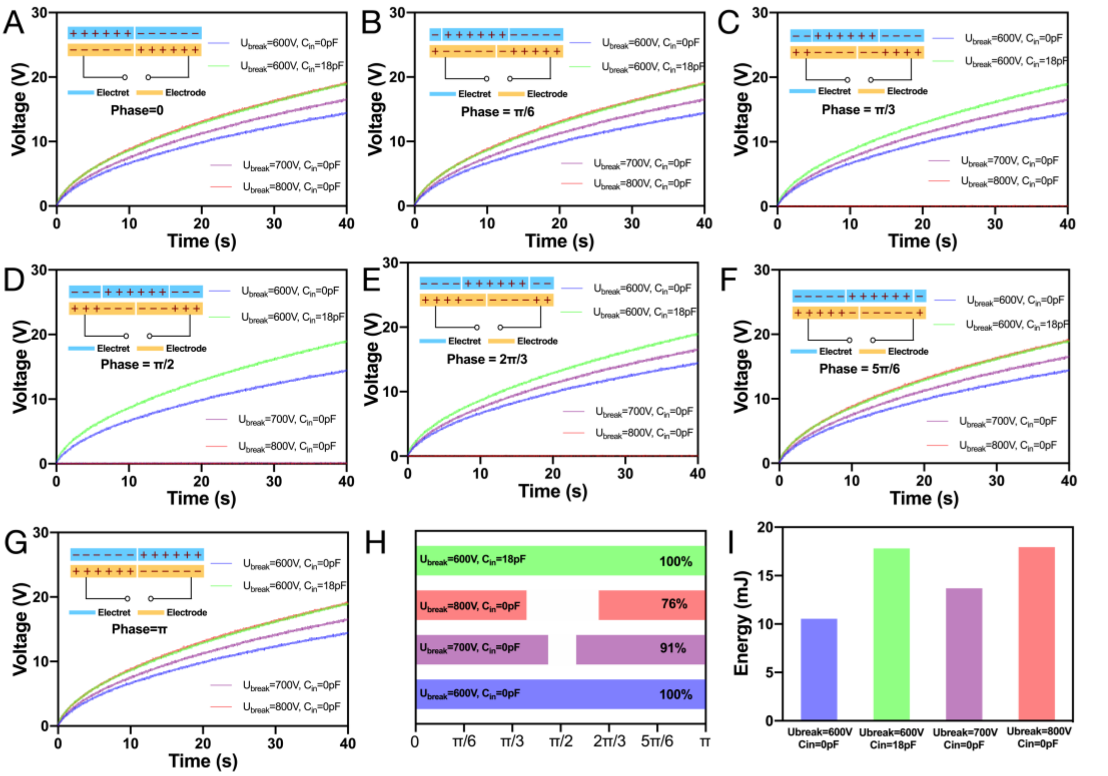

Figure S12. Effects of φ on the charging performance of EMU with different U_{break} and C_{in}. (A-G) Measured charging curves with C_s = 100 µF under different φ, U_{break}, C_{in}. (H) Range of φ that SST can be turned on, obtained by simulation as described in Note S1. (I) Stored energy on C_s in 40s under different U_{break} and C_{in} at $\varphi = 0$.

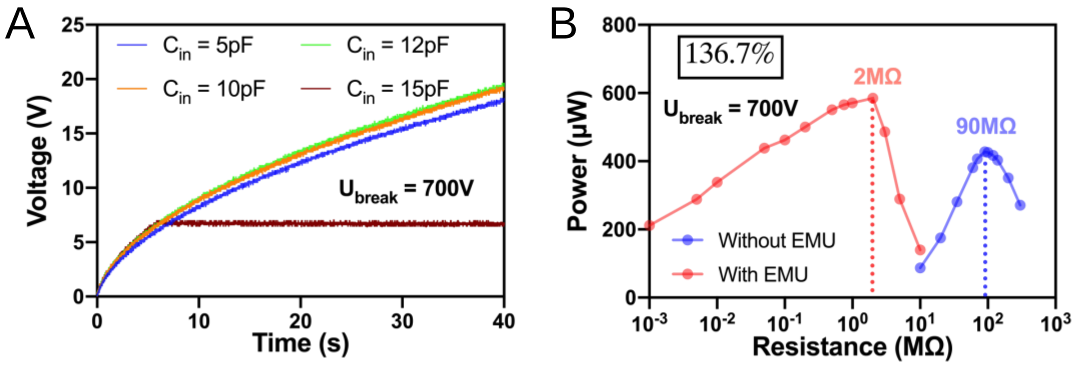

Figure S13. Measurement results of swREG with EMU under U_{break} = 700 V. (A)Charging curves with different C_{in}. (B) Power-load curves with C_{in} = 12 pF. With C_{in} = 15 pF, the voltage on C_s stopped rising at about 7V, since the voltage across SST becomes lower than U_{break} as the voltage on C_s reaches 7 V, resulting in SST cannot be turned on.

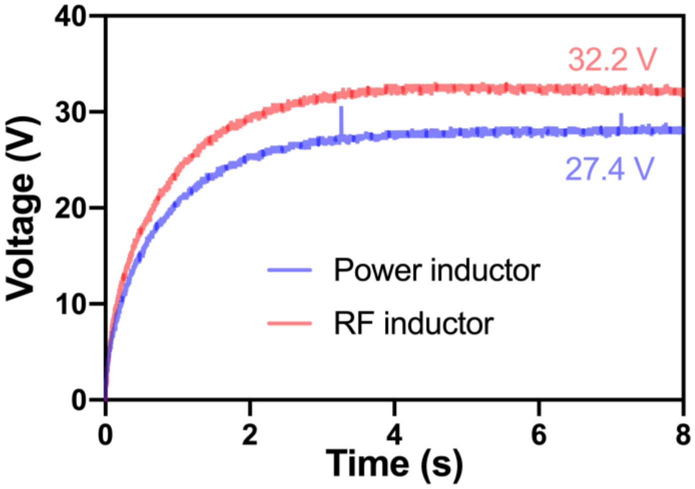

Figure S14. Voltage on the load of 2 MΩ under power inductor and RF inductor with U_{break} = 600 V, C_{in} = 18 pF in continuous mode.

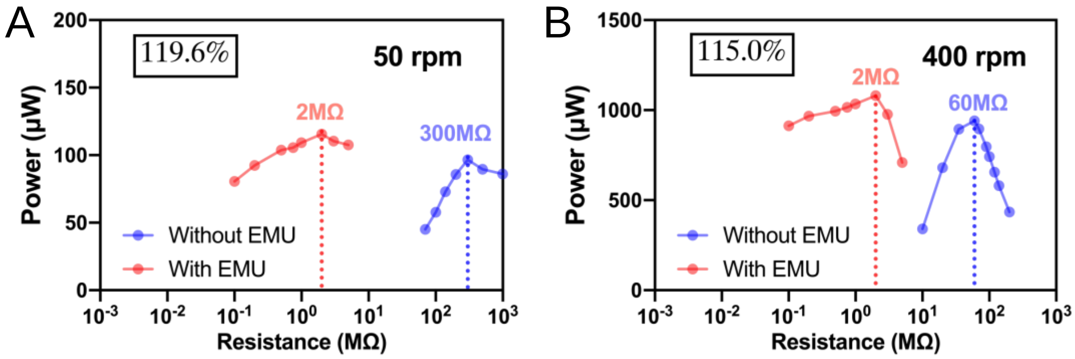

Figure S15. Power-load curves of swREG with U_{break} = 600V, C_{in} = 18 pF in continuous mode under different rotation speeds. (A) Under 50 rpm. (B) Under 400 rpm.

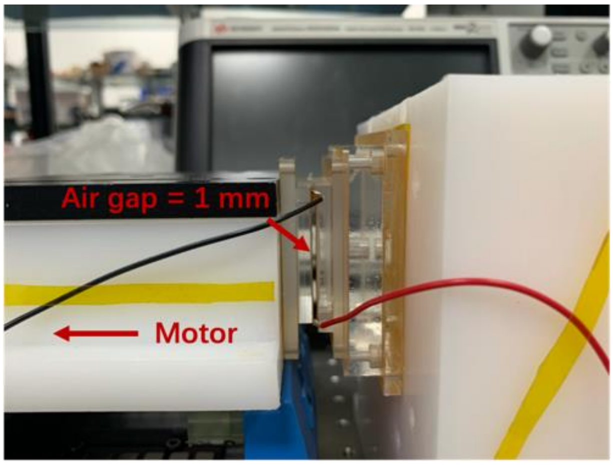

Figure S16. The photo of the fabricated CS-TENG.

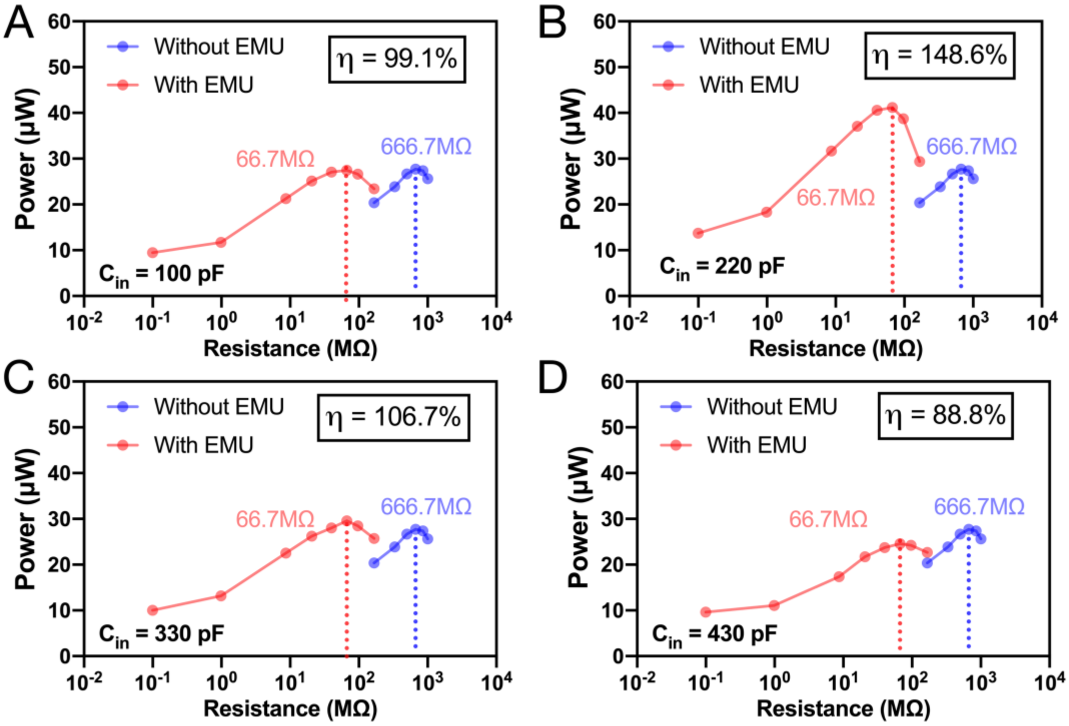

Figure S17. Power-load curves of CS-TENG under different C_{in} with and without EMU. (A) C_{in} = 100 pF. (B) C_{in} = 220 pF. (C) C_{in} = 330 pF. (D) C_{in} = 430 pF.

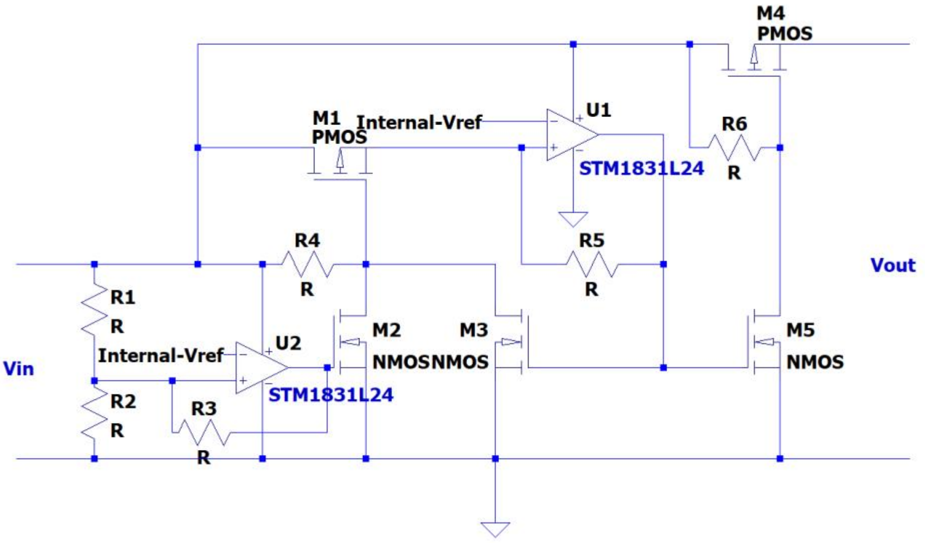

Figure S18. Circuit schematic of the trigger.

Note.S1 Circuit model and parameters in simulations

The simulations in this paper are based on LT-SPICE with a circuit mode of a square wave current source paralleled with generator's capacitor that was verified in our previous work [S11]. The circuit model used in Figure 2C&E is shown in Figure S19A, whose external loads are 1000 TΩ and 10 GΩ, respectively. The circuit model used in Figure S2 is the same to Figure 2C. The circuit model used in Figure 2D and Figure S12H is shown in Figure S19B. Without special instructions, the parameters used in the simulation are the default values in Table. S3. Here, *Sw* in FigureS19B, which serves as a spark switch, is a voltage-controlled switch controlled by the voltage between Node a and Node b, and its turn-on voltage is set as the peak voltage at each φ of Figure 2C. For Figure S12H, the turn-on voltage is set as each U_{break}, and the amplitude of the current source is set as 2.6 μA for U_{oc} = 650 V.

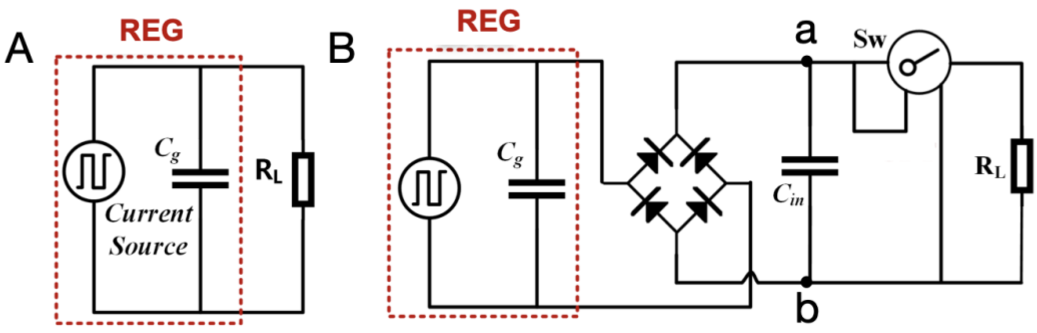

Figure S19. Circuit models used in simulations. (A) For Figure 2C&E and Figure S2. (B) For Figure 2D and Figure S12H.

Table S3. Default parameters of the simulation models of REG and swREG

Amplitude of current source (I_{sc})	2.4 μA
Generator capacitance (C_g)	25 pF
Turn-off voltage of the switch (U_{off})	0 V
On-resistance of the switch (R_{on})	0.1 Ω
Off-resistance of the switch (R_{off})	1 TΩ
Working period pf REG (T_{REG})	25 ms

Note.S2 Power enhancement of swREG

When $U_{break} = U_{oc}$ and $C_{in} = C_g$, the accumulated energy of swREG in a working period is

$$E_{in} = \frac{1}{2}(C_g + C_{in})U_{break}^2 = C_g U_{oc}^2 \tag{S1}$$

Then the maximum average power of swREG is

$$P_{av-max} = \frac{C_g U_{oc}^2}{T} \tag{S2}$$

where T is the working period of swREG, half of that of REG. From our previous work [S11], the maximum average power of REG under the matched load without EMU is

$$P_{woEMU} = 0.104 * \frac{16 C_g U_{oc}^2}{\pi T} \tag{S3}$$

From Eq. (S2) and Eq. (S3),

$$\frac{P_{av-max}}{P_{woEMU}} = 1.89 \tag{S4}$$

Note.S3 True value of U_{oc}

In practice, due to the input resistance and parasitic capacitance of the measurement circuit for U_{oc}, the measured U_{oc} is less than its true value. Here, by integrating the absolute value of short-circuit current of REG in Figure S6B, we can obtain the charges on the electret σS of 34.35 nC. According to $U_{oc} = \sigma S/2C_g$ with C_g = 25 pF, we can obtain the actual U_{oc} of 687 V, which is higher than U_{break} = 600 V.

Note.S4 Comparison of two kinds of inductors

The measurement circuit for the comparison of two kinds of inductors is shown in Fig. S20A, which consists of a DC voltage source with the output of 1000 V, a resistor of 1 GΩ, a capacitor C_t = 25 pF, a ceramic SST with U_{break} = 700 V, and a buck converter. Here, the RF inductors are from the series of 07MFG, Fastron, and the power inductors are from the series of RSF1314, Coilcraft. Figure S20B shows the charging curves on C_s = 10 μF under the RF inductor and power inductor of 1 mH. And Figure 20C&D show the charging curves on C_s = 100 μF under EMU with RF inductors and power inductors of different inductance, respectively. Obviously, the performance of EMU with the RF inductor is better than that with the power inductor, probably because the power inductor cannot respond fast enough to the high switching speed of SST, resulting in relatively higher loss than RF inductors. And overall, the inductance has little effect on the performance of EMU. While the performance of EMU with the RF inductor of 1mH is slightly lower than that with the other RF inductors. This may be because the RF inductor of 1mH cannot store the total energy from C_t. Figure S20E compares the charging curves on C_s = 10 μF with C_t = 200 pF under EMU with the RF inductors of 1mH from the series of 07MFG and 11PHC (for high current), respectively. Obviously, for C_t larger, namely, more energy, the RF inductor of 11PHC is better. Thus, we subsequently used inductors of 07MFG and 11PHC for RFEG and CS-TENG, respectively.

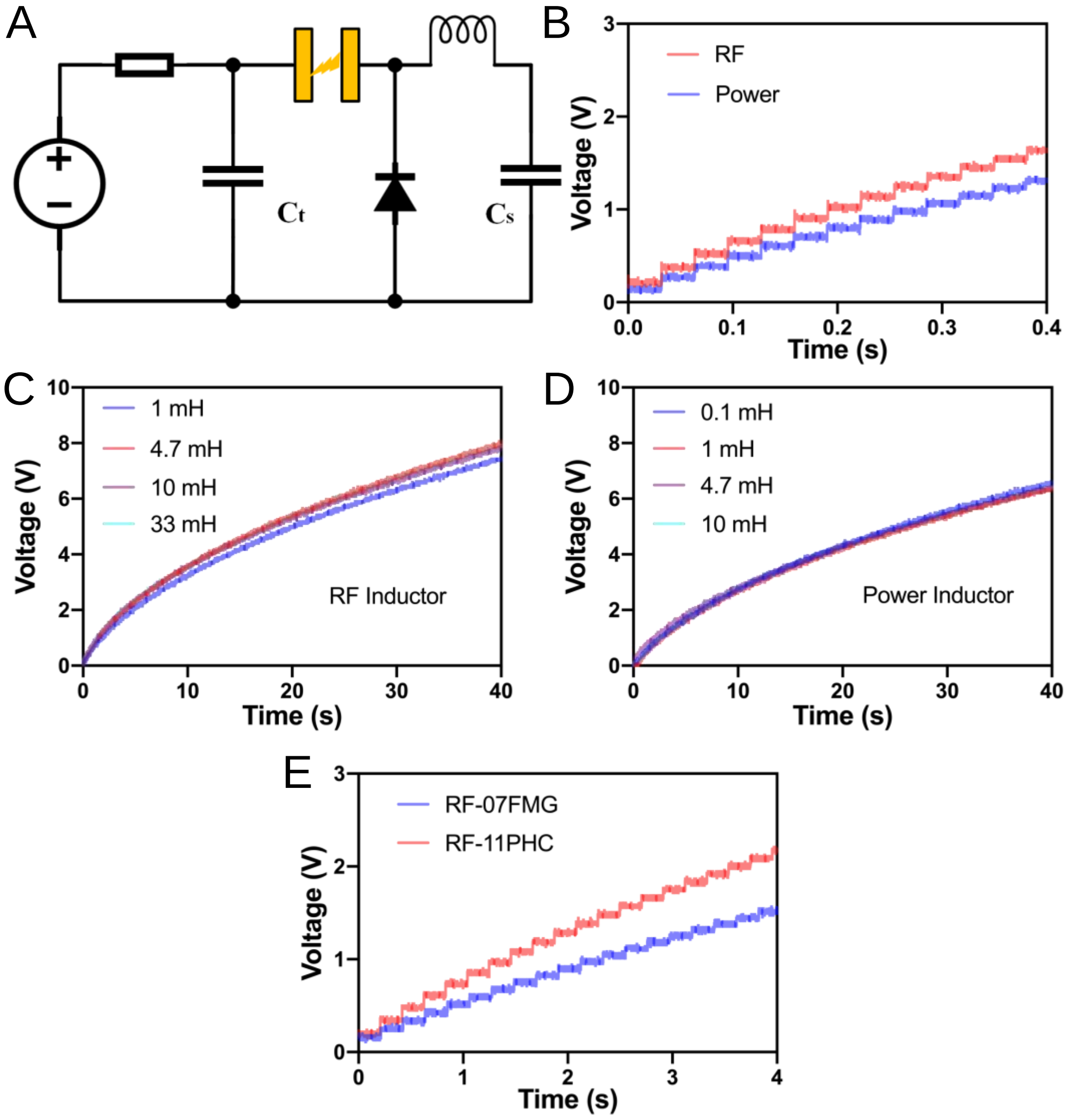

Figure S20. Comparison of two kinds of inductors. (A) Schematic of the measurement circuit. (B) The comparison of the charging curves on C_s = 10 μF under RF inductor and power inductor of 1 mH. (C & D) The charging curves on C_s = 100 μF under EMU with RF inductor and power inductor of different inductance, respectively. (E) The charging curves under two kinds of RF inductors with C_t = 200 pF.

Note.S5 Charging efficiency curve under EMU

From Figure 4B, the energy transfer efficiency of EMU from C_g and C_{in} to C_s can be calculated as:

$$\eta_e(t) = \frac{\frac{1}{2} \times C_s \times (U_t^2 - U_{t-1}^2)}{\frac{1}{2} \times (C_g + C_{in}) U_{break}^2 \times k}$$

Where U_t and U_{t-1} are the voltage on C_s at t and t-1, respectively. k is the number of discharges between t and t-1, here k = 80 (1 second). When C_{in} = 18 pF and U_{break} = 600 V, the charging efficiency curve is shown in Fig. S21. After 5s, the charging efficiency keeps

stable, and is about 85%.

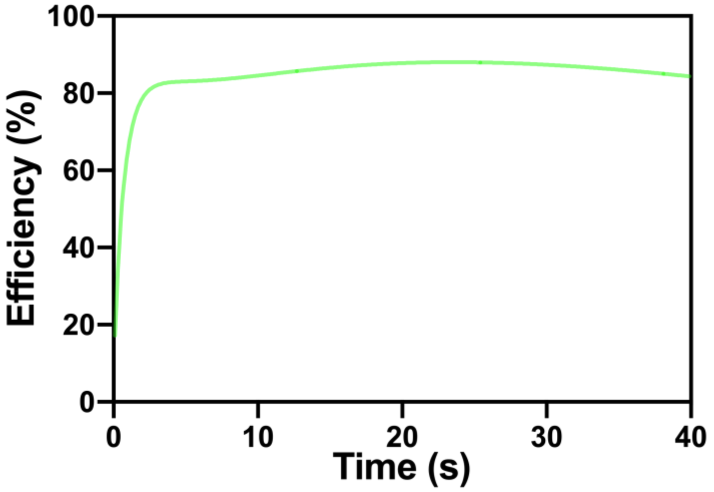

Figure S21. The charging efficiency curve with C_{in} = 18 pF and U_{break} = 600 V.

Reference:

[S1] Z. Cao, S. Wang, M. Bi, Z. Wu, X. Ye, Largely enhancing the output power and charging efficiency of electret generators using position-based auto-switch and passive power management module, Nano Energy. 66 (2019) 104202. https://doi.org/https://doi.org/10.1016/j.nanoen.2019.104202.

[S2] W. Yan, Y. Liu, P. Chen, L.N.Y. Cao, J. An, T. Jiang, W. Tang, B. Chen, Z.L. Wang, Flexible Film-Discharge-Switch Assisted Universal Power Management System for the Four Operation Modes of Triboelectric Nanogenerators, Adv. Energy Mater. 12 (2022) 2103677. https://doi.org/10.1002/AENM.202103677.

[S3] X. Cheng, L. Miao, Y. Song, Z. Su, H. Chen, X. Chen, J. Zhang, H. Zhang, High efficiency power management and charge boosting strategy for a triboelectric nanogenerator, Nano Energy. 38 (2017) 438–446. https://doi.org/https://doi.org/10.1016/j.nanoen.2017.05.063.

[S4] Z. Wang, W. Liu, W. He, H. Guo, L. Long, Y. Xi, X. Wang, A. Liu, C. Hu, Ultrahigh Electricity Generation from Low-Frequency Mechanical Energy by Efficient Energy Management, Joule. 5 (2021) 441–455. https://doi.org/10.1016/J.JOULE.2020.12.023.

[S5] F. Xi, Y. Pang, W. Li, T. Jiang, L. Zhang, T. Guo, G. Liu, C. Zhang, Z.L. Wang, Universal power management strategy for triboelectric nanogenerator, Nano Energy. 37 (2017) 168–176. https://doi.org/https://doi.org/10.1016/j.nanoen.2017.05.027.

[S6] Z. Wang, Q. Tang, C. Shan, Y. Du, W. He, et al. Giant performance improvement of triboelectric nanogenerator systems achieved by matched inductor design, Energy & Environmental Science, 14(12), 6627-6637.

[S7] W. Harmon, D. Bamgboje, H. Guo, T. Hu, et al. Self-driven power management system for triboelectric nanogenerators, *Nano Energy*, *71*, 104642.

[S8] Z. Cao, Z. Wu, R. Ding, S. Wang, Y. Chu, J. Xu, J. Teng, X. Ye, A compact triboelectric nanogenerator with ultrahigh output energy density of 177.8 J m−3 via retarding air breakdown, Nano Energy. 93 (2022) 106891. https://doi.org/https://doi.org/10.1016/j.nanoen.2021.106891.

[S9] W. Liu, Z. Wang, G. Wang, Q. Zeng, W. He, L. Liu, X. Wang, Y. Xi, H. Guo, C. Hu, Switched-capacitor-convertors based on fractal design for output power management of triboelectric nanogenerator, Nat. Commun. 11 (2020) 1–10.

[S10] Y. Gao, D. Liu, Y. Li, J. Liu, L. Zhou, X. Li, Z. Zhao, S. Li, P. Yang, Z.L. Wang, J. Wang, Achieving high-efficient triboelectric nanogenerators by suppressing electrostatic breakdown effect, Energy Environ. Sci. (2023). https://doi.org/10.1039/D3EE00220A.

[S11] M. Bi, S. Wang, X. Wang, X. Ye, Freestanding-electret rotary generator at an average conversion efficiency of 56%: Theoretical and experimental studies, Nano Energy. 41 (2017) 434–442. https://doi.org/10.1016/j.nanoen.2017.09.057.

[S12] Y. Zi, S. Niu, J. Wang, Z. Wen, W. Tang, Z.L. Wang, Standards and figure-of-merits for quantifying the performance of triboelectric nanogenerators, Nat. Commun. 6 (2015) 8376.
